# Human Herpesvirus 8 Seropositivity Among Sexually Active Adults in Uganda

**DOI:** 10.1371/journal.pone.0021286

**Published:** 2011-06-21

**Authors:** Fatma M. Shebl, Sheila C. Dollard, Ruth M. Pfeiffer, Benon Biryahwaho, Minal M. Amin, Stella S. Munuo, Wolfgang Hladik, Ruth Parsons, Barry I. Graubard, Sam M. Mbulaiteye

**Affiliations:** 1 Division of Cancer Epidemiology and Genetics, National Cancer Institute, Bethesda, Maryland, United States of America; 2 Centers for Disease Control and Prevention, Atlanta, Georgia, United States of America; 3 Uganda Virus Research Institute, Entebbe, Uganda; 4 Information Management Services, Rockville, Maryland, United States of America; 5 Centers for Disease Control and Prevention, Entebbe, Uganda; University of Nebraska - Lincoln, United States of America

## Abstract

**Introduction:**

Sexual transmission of human herpesvirus 8 (HHV8) has been implicated among homosexual men, but the evidence for sexual transmission among heterosexual individuals is controversial. We investigated the role of sexual transmission of HHV8 in a nationally representative sample in Uganda, where HHV8 infection is endemic and transmitted mostly during childhood.

**Materials and Methods:**

The study population was a subset of participants (*n* = 2681) from a population-based HIV/AIDS serobehavioral survey of adults aged 15–59 years conducted in 2004/2005. High risk for sexual transmission was assessed by questionnaire and serological testing for HIV and herpes simplex virus 2. Anti-HHV8 antibodies were measured using two enzyme immunoassays targeting synthetic peptides from the K8.1 and orf65 viral genes. The current study was restricted to 2288 sexually active adults. ORs and 95% CIs for HHV8 seropositivity were estimated by fitting logistic regression models with a random intercept using MPLUS and SAS software.

**Results:**

The weighted prevalence of HHV8 seropositivity was 56.2%, based on 1302 seropositive individuals, and it increased significantly with age (*P*_trend_<0.0001). In analyses adjusting for age, sex, geography, education, and HIV status, HHV8 seropositivity was positively associated with reporting two versus one marital union (OR:1.52, 95% CI: 1.17–1.97) and each unit increase in the number of children born (OR: 1.04, 95% CI: 1.00–1.08), and was inversely associated with ever having used a condom (OR: 0.64, 95% CI: 0.45–0.89). HHV8 seropositivity was not associated with HIV (*P* = 0.660) or with herpes simplex virus 2 (*P* = 0.732) seropositivity. Other sexual variables, including lifetime number of sexual partners or having had at least one sexually transmitted disease, and socioeconomic variables were unrelated to HHV8 seropositivity.

**Conclusion:**

Our findings are compatible with the conclusion that sexual transmission of HHV8 in Uganda, if it occurs, is weak.

## Introduction

Human herpesvirus 8 [HHV8, also called Kaposi sarcoma (KS)-associated herpesvirus or KSHV] is the cause of KS [1], and it is thought to be transmitted sexually among homosexual men [2]. In the United States, HHV8 seroprevalence is 15–40 times higher in homosexual men [3] than in the general population [4]. The role of heterosexual transmission of HHV8, especially in sub-Saharan Africa, where infection is endemic [5], [6], is less clear [7]. Transmission appears to occur horizontally during childhood [8], [9], [10], mostly in the family setting [10], [11]. Age-related increase in HHV8 seropositivity, reported in many [12], [13], [14], but not all studies [15], has been interpreted as suggesting sexual transmission of HHV8. Other indirect evidence includes higher HHV8 seroprevalence in men than women [12], [16].

Association of HHV8 seropositivity with specific sexual exposures has been reported, but the results are not consistent within and between studies [7]. In Lagos, Nigeria, HHV8 seropositivity was two times higher among commercial sex workers (CSWs) and in men and women with evidence of any sexually transmitted disease (STD), compared with low-risk adults drawn from the general population [17]. In that study, the association of HHV8 seropositivity with a positive laboratory test of syphilis was significant in women, but not in men [17]. In Kenya, HHV8 seropositivity was higher among HIV-seronegative CSWs with gonorrhea compared with those without [18], and among long-distance drivers with syphilis compared with those without [19]. No association with HHV8 seropositivity was noted with number of sex partners, sex acts per week, condom use, *Trichomonas*, *Chlamydia*, vaginal candidiasis or bacterial vaginosis in the CSWs [18] and with HIV infection, having sex with prostitutes, urethral discharge or genital ulcer in the long-distance truck drivers [19]. No association was noted between HHV8 seropositivity and HIV, STDs, and number of sexual partners in two large studies; one of which looked at 2103 miners, CSWs, and adults in a township in South Africa [20], and another that looked at 1404 adults from a general population living in one district in Uganda [21]. These studies were conducted in high-risk populations [17], [18], [19], [20] or in general populations that were relatively homogeneous because they were recruited from a small geographical area [12], [21], [22], and their findings might not be generalizable. Understanding whether sexual transmission of HHV8 occurs is important for public health in sub-Saharan Africa [7] because both HHV8 and HIV, which is sexually transmitted, are highly prevalent [23], and KS has become the most common cancer in many countries during the AIDS epidemic [24]. We have reported previously that HHV8 seropositivity was higher in men than in women, increased with age, and varied geographically [16] in a nationally representative cohort that was established to investigate sexual risk factors for HIV infection in Uganda [25], [26]. In the current paper, we investigated the association between sexual risk factors with HHV8, adjusting for sex, age and geography among sexually active adults from the same cohort.

## Methods

### Ethics statement

Ethical approval to conduct the Uganda HIV/AIDS serobehavioral survey (UHSBS) in 2004/2005 was obtained from the Uganda Virus Research Institute (UVRI) Science Ethics Committee and the National Council of Science and Technology in Uganda and from the Centers for Disease Control and Prevention (CDC, Atlanta, Georgia, USA) [16], [25], [26]. Written informed consent was obtained from the participant before a questionnaire was administered and venous blood was removed for HIV, syphilis, herpes simplex virus 2 (HSV2), and hepatitis B virus (HBV) serology [25]. Blood was stored for use in future studies for participants who consented to this. Because we used samples delinked from individual identifiers, ethical approval to test the samples for HHV8 serology was obtained from the Office of Human Subject Research at the National Institutes of Health.

### Study population

The study population for HHV8 was a subset of the UHSBS cohort. UHSBS was designed to obtain accurate estimates of risk factors for HIV/AIDS among Ugandans aged 15–59 years. The cohort was recruited using a two-stage, non-stratified cluster design [16], [25], [26]. The first stage involved obtaining a random sample of enumeration areas, primary sampling units (PSUs), from the 2002 Uganda National Census enumeration area list [27] for a sample size of 417 PSUs, including 74 from urban areas and 343 from rural areas. The second stage involved obtaining a random sample of 25 households from each PSU for a sample size of 10,425 households [25], [26]. An additional 12 households found during the survey were included. A total of 19,656 adults in the selected households were interviewed, for a response rate of 92.0%. The interview elicited detailed individual and household information. Individual information included age, sex, residence, religion, formal education, occupation, age of sexual debut, marital status and number of marital unions, lifetime number of sexual partners, casual sexual contacts, condom use, circumcision status for men, and history of STD. Household information included ownership of durable goods, such as a car, bike, telephone, or radio, and the condition of the dwelling unit, the material used to construct the floor (dirt or cement), and the source of drinking water (surface, public or private tap). Data on household assets and household characteristics were used to construct a standardized wealth index, using principle components analysis, and levels categorized using quintiles [26]. A venous blood sample was obtained from consenting participants, for a response rate of 86% [25]. HIV, syphilis, HSV2 and HBV serology was performed using commercial assays.

The HHV8 study used a random sample of 2681 individuals selected from the 19,656 individuals who participated in the UHSBS 2004/2005 and tested for HHV8 serologically [16]. The current study was restricted to sexually active subjects, based on a “yes” answer to the question “have you ever had sex” on the UHSBS questionnaire.

### HHV8 serological testing

Anti-HHV8 antibodies were assayed at the UVRI HIV Reference Laboratory, Entebbe, Uganda using enzyme immunoassays based on synthetic peptides encoded by K8.1 and orf65 viral genes [28], [29], as reported previously [16]. Initial pilot studies conducted at UVRI in Uganda and at the CDC in the United States showed that large-scale HHV8 testing in Uganda is feasible [16]. Peptides were manufactured at the CDC Herpes Virology Laboratory and sent to Uganda immediately before testing. Blank uncoated wells were run as negative controls for each plasma specimen to provide background optical density (OD) readings. HHV8 test sample OD readings were adjusted by subtracting the OD reading of the negative well from the test sample OD readings. The OD values for the blank wells were subtracted from the OD values of the test samples to obtain adjusted OD values. The samples were categorized as HHV8 seropositive when the adjusted OD values for K8.1 were >0.7, when the K8.1 value was 0.5–0.7 and the orf65 was >2.5; samples with K8.1 value 0.5–0.7 and orf65 ≤2.5 were categorized as indeterminate. Samples that did not meet these criteria were categorized as negative.

### Statistical methods

Crude associations for main effects of sexual socioeconomic and environmental factors with HHV8 seropositivity were assessed using SURVEYFREQ in SAS version 9.2., (SAS Institute, Cary, NC, USA) separately for men and women, and then for both groups combined. Weights and cluster sampling were accounted for when calculating *P* values using the Rao–Scott χ^2^ method [30]. The ORs of association between sexual, socioeconomic, and environmental characteristics with HHV8 seropositivity were estimated by fitting logistic regression models with random intercepts, with individuals *i* nested within PSUs *j* at level 2, to account for the correlation between individuals from the same PSU, using MPLUS [31]. The random intercept was assumed to have a normal distribution with a mean of zero and a variance *Ψ*, and the observations made on individuals from different PSUs were assumed to be independent of each other. New weights were derived by re-scaling the survey weights as described by Pfefferman *et al.* [32], and incorporated in the logistic regression models to account for the survey design. Multivariable logistic regression models were constructed by including variables that were associated with HHV8 seropositivity at *P*<0.05 in the bivariate analysis. PSU-level variables (average age and percentage with high school education for each sampled PSU) and individual-level variables (geographical region of residence), which we have previously shown to be associated with HHV8 seropositivity [16], as well as HIV status were included in the multivariable models as potential confounders. Wald tests were calculated using the sandwich variance estimation to obtain *P* values for hypothesis testing and to construct 95% CIs for the ORs. Residual intraclass correlation of HHV8 seropositivity among subjects in the same PSU [31] was estimated and the significance of the random effect (*Ψ* = 0 versus *Ψ*>0) was assessed using the likelihood-ratio test [31]. Two-sided *P*<0.05 was considered to be statistically significant.

## Results

The study included 2288 (85% of the HHV8 cohort) individuals who were sexually active, including 1026 (44.6%) men and 1262 (45.4%) women. The mean age was 32.2 (95% CI: 31.7–32.7) years. Most resided in rural areas (85%) and 84.1% reported at least one marital union.

The weighted HHV8 seropositivity was 56.2%, based on 1302 HHV8 seropositive individuals. The weighted HHV8 seropositivity for men was not significantly different from that for women (58.0% vs. 54.6%, *P* = 0.114). HHV8 seropositivity increased from 56.6% in men aged 15–19 years to 71.5% in those aged 50–59 years (*P* = 0.03) and from 46.2% in women aged 15–19 years to 80.4% in those aged 50–59 years (*P*<0.0001). In unadjusted analyses combining men and women, HHV8 seropositivity was positively associated with having ever been married and having two marital unions (*P*<0.0001). Conversely, HHV8 seropositivity was inversely associated with having ever used a condom or using a condom at the last time of sexual intercourse (*P*<0.001) (Table 1). HHV8 seropositivity was inversely associated with ownership of a radio, car, bike, or mobile telephone, durable or expensive home goods, and high quintiles of wealth index. Living in a house with a floor constructed from mud or dirt was associated with increased HHV8 seropositivity (Table 2).

**Table 1:** Crude association between HHV8 seropositivity and demographical and sexual variables among sexually active adults in Uganda.

Characteristics	Men		Women		Men and women combined	
	n/N (%)^a^	p value^b^	n/N (%)^a^	p value^b^	OR (95% CI)	p value^b^
**All subjects**	609/1026 (58.03)	NA	693/1262 (54.64)	NA	1.15 (0.97,1.36)	0.114
**Age groups, years**		0.03		<0.0001		<0.0001
15–19	76/131 (56.63)		59/136 (46.24)		Reference	
20–29	176/320 (53.74)		247/488 (50.06)		1.12 (0.90,1.40)	
30–39	170/281 (58.99)		188/349 (53.53)		1.29 (1.00,1.65)	
40–49	104/178 (56.44)		122/193 (60.51)		1.43 (1.10, 1.86)	
50–59	83/116 (71.52)		77/96 (80.43)		3.21 (2.21,4.67)	
**Sexual risk factors**						
Ever married		0.12		0.03		0.027
No	133/244 (53.5)		52/125 (44.4)		Reference	
Yes	476/782 (59.4)		641/1,137 (55.8)		1.32 (1.03,1.69)	
Number of unions		0.01		<0.001		<0.0001
1	221/395 (55.3)		469/868 (53.1)		Reference	
2	159/237 (65.4)		171/266 (64.4)		1.59 (1.30,1.94)	
Total lifetime partners						
1 to 2	93/155 (58.4)		305/524 (57.3)		Reference	
3 to 4	225/397 (55.3)		323/624 (52.0)		0.84 (0.69,1.03)	
5+	262/428 (59.6)		62/105 (60.0)		1.09 (0.86,1.39)	
*P for trend*		0.53		0.56		0.54
Paid/received money for sex in last 12-month		0.16		<0.0001		0.24
No	25/53 (43.2)		0/1 (--.-)		Reference	
Yes	8/12 (64.4)		3/6 (44.6)		1.86 (0.64,5.52)	
Ever had STD^c^						
No	474/805 (57.6)	0.45	479/880 (53.6)	0.33	Reference	0.32
Yes	135/220 (59.7)		214/382 (56.8)		1.10 (0.91,1.33)	
HIV		0.72		0.82		0.96
No	577/971 (58.2)		647/1,177 (54.6)		Reference	
Yes	32/54 (55.9)		46/84 (56.0)		0.99 (0.69,1.43)	
HSV2		0.20		0.25		0.14
No	338/588 (56.4)		314/585 (52.8)		Reference	
Yes	271/436 (60.3)		378/673 (56.3)		1.14 (0.96,1.36)	
HBV		0.83		0.35		0.29
No	167/285 (57.6)		173/360 (49.1)		Reference	
Yes	30/50 (59.2)		12/19 (60.8)		1.32 (0.79,2.20)	
Ever used condoms		<0.001		0.02		<0.001
No	352/548(62.9)		524/885 (58.4)		Reference	
Yes	122/235 (50.7)		95/195 (48.9)		0.66 (0.52,0.84)	
Used condom in last intercourse		0.07		0.08		0.02
No	454/754 (58.5)		517/933 (54.5)		Reference	
Yes	69/138 (50.3)		40/97 (43.5)		0.70 (0.52,0.95)	
Circumcised		0.11		NA		0.11
No	435/752 (56.5)		NA		Reference	
Yes	174/273 (62.7)		NA		1.29 (0.94,1.78)	

a Weighted frequency of HHV8 seropositivity.

b *P* for heterogeneity unless otherwise is indicated.

c Based on questionnaire data.

**Table 2:** Crude association between HHV8 seropositivity and socioeconomic and environmental variables among sexually active adults in Uganda.

Characteristics	Men		Women		Men and women combined	
	n/N (%)^a^	p value^b^	n/N (%)^a^	p value^b^	OR (95% CI)	p value^b^
**Socioeconomic exposures**						
Ownership: car, bike, radio, telephone		<0.01		0.01		<0.001
No	190/285 (65.3)		237/385 (59.3)		Reference	
Yes	521/926 (55.3)		556/1,082 (51.8)		0.69 (0.57,0.85)	
Durable/expensive home goods		0.05		<0.01		0.001
No	179/278 (63.4)		212/336 (61.2)		Reference	
Yes	532/933 (56.1)		581/1,131 (51.8)		0.69 (0.55,0.87)	
Wealth index quintile score		0.01		0.11		0.03
Poorest	144/222 (63.3)		176/279 (61.4)		Reference	
Poorer	151/236 (63.1)		174/309 (54.6)		0.80 (0.61,1.06)	
Middle	133/233 (56.8)		134/253 (52.9)		0.70 (0.52,0.95)	
Richer	127/231 (54.1)		142/275 (51.9)		0.66 (0.49,0.90)	
Richest	157/292 (52.8)		167/351 (49.5)		0.64 (0.48,0.85)	
*P for trend*		<0.01		<0.001		0.002
**Environmental factors**						
Any child <17 years old		0.80		0.70		0.87
No	96/164 (57.1)		46/86 (52.6)		Reference	
Yes	513/862 (58.2)		647/1,176 (54.8)		1.02 (0.77,1.35)	
Live stock		0.16		0.68		0.59
No	208/333 (61.6)		221/421 (53.6)		Reference	
Yes	400/687 (56.7)		470/837 (55.1)		0.95 (0.78,1.6)	
Mud or dirt floor material		0.01		0.04		0.003
No	123/240 (50.2)		131/280 (49.4)		Reference	
Yes	486/781 (61.0)		558/973 (56.2)		1.42 (1.13,1.79)	
Water source		0.32		0.55		0.27
Private	323/549 (57.7)		394/726 (54.1)		Reference	
Public	128/228 (55.1)		144/271 (53.9)		0.95 (0.77,1.18)	
Surface	158/247 (62.0)		155/264 (57.0)		1.17 (0.93,1.46)	

a Weighted frequency of HHV8 seropositivity.

b *P* for heterogeneity unless otherwise is indicated.

Similar patterns of association of HHV8 seropositivity with sexual, socioeconomic, and environmental variables were observed in sex-specific analyses, with slight differences. For example, HHV8 seropositivity was related to marital status and paying or receiving money for sex in women, but not in men (Table 1). Conversely, HHV8 seropositivity showed significant heterogeneity with wealth index in men, but not in women, although a trend for decreasing positivity with increasing wealth index was observed in men and women (Table 2).

In multivariable models, adjusting for age and education as cluster-level variables, HHV8 seropositivity was positively associated with two versus one marital union (OR: 1.52, 95% CI: 1.17–1.97), each unit increase in children born (OR: 1.04, 95% CI: 1.00–1.08), and was inversely associated with ever having used a condom (OR: 0.64, 95% CI: 0.45–0.89) (Table 2). HHV8 seropositivity was unrelated to HIV (*P* = 0.660), HSV2 (*P* = 0.732), and socioeconomic factors (Table 3). About 21.4% of variance of HHV8 seropositivity, which was correlated with PSUs (or residual intraclass correlation), remained unaccounted for by variables included in the final model (*P* = 0.001).

**Table 3:** Adjusted association between HHV8 seropositivity and selected risk factors among sexually active adults in Uganda.

Characteristic	Men		Women		Men and Women Combined	
	OR (95% CI)^a^	*P* value	OR (95% CI)^a^	*P* value	OR (95% CI)^a^	*P* value
**Sexual risk factors**						
Number of unions	1.76 (1.17–2.66)	0.007	1.46 (1.03–2.08)	0.035	1.52 (1.17–1.97)^b^	0.002
HIV	0.85 (0.35–2.04)	0.712	1.24 (0.66–2.33)	0.496	1.12 (0.67–1.90)	0.660
HSV2	0.92 (0.63–1.35)	0.679	1.06 (0.77–1.46)	0.724	1.05 (0.81–1.34)	0.732
Ever used condoms	0.60 (0.37–0.98)	0.042	0.70 (0.46–1.07)	0.101	0.64 (0.45–0.89)	0.008
**Socioeconomic exposures**						
Ownership: car, bike, radio, telephone	0.758 (0.46–1.24)	0.258	0.89 (0.61–1.31)	0.557	0.86 (0.63–1.18)	0.354
Durable/expensive home goods	0.98 (0.56–1.73)	0.951	0.80 (0.52–1.22)	0.297	0.83 (0.59–1.18)	0.309
Wealth quintile score index						
Poorest	Reference		Reference		Reference	
Poorer	0.93 (0.50–1.72)	0.804	0.84 (0.52–1.34)	0.454	0.83 (0.57–1.21)	0.343
Middle	0.75 (0.38–1.51)	0.426	0.79 (0.48–1.28)	0.334	0.76 (0.50–1.17)	0.209
Richer	0.73 (0.34–1.58)	0.425	0.87 (0.50–1.51)	0.614	0.79 (0.50–1.26)	0.326
Richest	0.94 (0.29–3.09)	0.921	1.18 (0.52–2.70)	0.695	0.98 (0.51–1.91)	0.960
**Environmental factors**						
Total children ever born	1.00 (0.94–1.06)	0.973	1.07 (1.02–1.12)	0.003	1.04 (1.00–1.08)^c^	0.037
Mud or dirt floor material	1.81 (0.71–4.60)	0.218	1.40 (0.72–23.69)	0.319	1.49 (0.85–2.59)	0.162
Water source						
Private	Reference		Reference		Reference	
Public	0.71 (0.23–2.13)	0.535	1.40 (0.54–3.60)	0.497	0.97 (0.46–2.04)	0.944
Surface	0.95 (0.29–3.11)	0.930	1.30 (0.47–3.61)	0.620	1.03 (0.46–2.29)	0.950
Age^d^	0.98 (0.88–1.10)	0.777	1.01 (0.92–1.10)	0.912	0.98 (0.90–1.07)	0.682

a Model includes variables that were significant (*P*<0.05) in the unadjusted analysis along with individual-level variables of sex and region, and PSU-level variables of mean age and percentage with greater than higher education for the PSU.

b Per each unit increase in number of marital unions.

c Per each unit increase in total children born.

d Per year increase in PSU-level average age.

## Discussion

HHV8 seropositivity was positively associated with reporting multiple marital unions and with each unit increase in the total number of children born. Conversely, it was inversely associated with having ever used a condom. These associations were independent of HIV, HSV2 and of age, sex, and region of residence [16]. HIV and HSV2, both strong markers of sexual transmission, were unrelated to HHV8 seropositivity. We interpret these results as clues for a role of sexual transmission of HHV8 in Uganda, in accord with findings from several other studies [12], [13], [17], [18], [19].

The inverse association with condom use is not new. Beaten and co-workers [19] have reported that HHV8 seropositivity was 30% decreased among Kenyan truck drivers who reported using a condom compared with those who did not, which suggested that condom use might protect from HHV8 infection. This finding was not replicated in two other studies that evaluated the hypothesis among female prostitutes from Kenya [18] and adults with cancer at hospitals in Kampala, Uganda [33], which casts doubt on its validity. Our results obtained from a large geographically heterogeneous population-based study conducted in Uganda where endemicity both of HHV8 and KS is high but variable [16] suggest that condom use and/or behavior correlated with the tendency to use condoms may protect from HHV8 seropositivity.

We noted, similar to previous studies conducted inside [21], [22], [33] and outside Uganda [20], that HHV8 seropositivity was not associated with lifetime number of sexual partners, reporting a history of any STD, or a positive laboratory test for HIV and HSV2. These null findings with HHV8 contrast sharply with the strong, positive, and consistent associations demonstrated between HIV infection questionnaire and laboratory sexual risk factors that have been reported from this cohort [25], [26]. The null or ambiguous associations between HHV8 seropositivity and sexual variables suggest that sexual transmission of HHV8 in Uganda, if it occurs, is weak. The ambiguous HHV8 associations in our study and in the literature highlight the difficulty of investigating sexual transmission of HHV8 in populations where non-sexual HHV8 transmission predominates. HHV8 seropositivity in the cohort restricted to sexually active individuals was similar to that observed in the cohort when both sexually active and sexually naïve individuals were included (56.2% vs. 55.4%) [16]. In both analyses, HHV8 seropositivity increased significantly with age. The reasons for the age-related increase in HHV8 seropositivity are unclear to us, but they may include a cohort effect (older people who may have been exposed to higher HHV8 transmission risks during childhood), or HHV8 reactivation, which may cause sero-conversion or increase of anti-HHV8 antibody titers, as people age, or new infections.

We observed a small but significant association between HHV8 seropositivity with each unit increase in the total number of children born. This result may indicate a greater propensity for intra-familial HHV8 transmission in families with many children [12], or it may be due to residual confounding with age. HHV8 seropositivity was unrelated to wealth index score and to drinking water from a surface water source; both measures of socioeconomic status. Most HHV8 transmission in Uganda occurs during childhood [34]; therefore, assuming that most HHV8 infections in our sample occurred during childhood, and that only a minority occurred during adulthood, this impedes our ability to find small, albeit, significant associations between current socioeconomic status and adult HHV8 transmission. Our fully adjusted model did not account for all variation in HHV8 seropositivity. About 20% of variance in HHV8 seropositivity was correlated with geographical clusters. This correlation indicates proneness to HHV8 seropositivity among individuals living in some geographical clusters. Some of us have speculated that geographical clustering of HHV8 seropositivity, which is observed at the macro [5] and micro level [16], may be due to helminthic parasites. Co-infection with parasites may influence HHV8 transmission by modulating host immunity [35] and viral control and shedding among seropositive individuals [36], or susceptibility at low levels of HHV8 exposure among uninfected individuals.

We acknowledge some caveats about our data. First, the results were from a cross-sectional study and the temporal relationship between correlated variables cannot be determined. Second, HHV8 serological methods are imperfect [37]. We used two lytic HHV8 enzyme immunoassays, which have been used with reasonably high sensitivity and specificity in several other studies of HHV8 conducted in Uganda [5], [11], [38]. Our results are comparable with those from studies of individuals from regions where data are common to both studies [5], [16]. Errors in classification cannot be excluded, but they would have been random and biased the associations towards the null, thus, making it difficult to demonstrate small effects. The strengths of our study include access to a nationally representative sample with detailed questionnaire and laboratory results for STDs from a country with well-characterized KS epidemiology before the AIDS epidemic. Sexual behavior was documented meticulously and associations between sexual risk factors with prevalent and incident HIV infection suggest that sexual exposure was measured reliably [25], [26]. Detailed information about socioeconomic, geographical, and environmental confounders was available and was used to adjust for confounding more than is normally possible in epidemiological studies conducted in Africa.

In conclusion, we observed positive associations between HHV8 seropositivity with two marital unions and the number of children ever born, and an inverse association with condom use in a representative cohort of sexually active Ugandans. HIV and HSV2 were unrelated to HHV8 seropositivity. We interpret our results as indicating that sexual transmission of HHV8 in Uganda, if it occurs, is weak.
